# Transcriptomic Analysis Reveals the Beneficial Effects of Spermidine in an ALS Mouse Model

**DOI:** 10.3390/biom16040566

**Published:** 2026-04-10

**Authors:** Cristian Fiorucci, Marianna Nicoletta Rossi, Rachele Di Santo, Illari Salvatori, Silvia Scaricamazza, Stefano Giuliani, Olga Carletta, Ermes Filomena, Davide Laurenti, Roberto Mattioli, Luciana Mosca, Cristiana Valle, Alberto Ferri, Anna Maria D’Erchia, Manuela Cervelli

**Affiliations:** 1 Department of Sciences, University of Roma Tre, 00146 Rome, Italy; cristian.fiorucci@unicamillus.org (C.F.); mariannanicoletta.rossi@uniroma3.it (M.N.R.); rachele.disanto@uniroma3.it (R.D.S.); stefano.giuliani@ifo.it (S.G.); olga.carletta@uniroma3.it (O.C.); 2 Laboratory of Neurochemistry IRCCS-Fondazione Santa Lucia, 00179 Rome, Italy; i.salvatori@hsantalucia.it (I.S.); silviascaricamazza@gmail.com (S.S.); cristiana.valle@cnr.it (C.V.); alberto.ferri@cnr.it (A.F.); 3 Institute of Translational Pharmacology (IFT), National Research Council (CNR), 00133 Rome, Italy; 4 Gene Expression and Cancer Models Unit, Department of Research and Advanced Technologies, Translational Research Area, IRCCS Regina Elena National Cancer Institute, 00144 Rome, Italy; 5 Department of Biosciences, Biotechnology and Environment, University of Bari Aldo Moro, 70125 Bari, Italy; ermes.filomena@uniba.it (E.F.); annamaria.derchia@uniba.it (A.M.D.); 6 Department of Biochemical Sciences “A. Rossi Fanelli”, Sapienza University of Rome, 00185 Rome, Italy; davide.laurenti@uniroma1.it (D.L.); roberto.mattioli@uniroma1.it (R.M.); luciana.mosca@uniroma1.it (L.M.); 7 Laboratory of Biochemistry and Molecular Biology, Department of Movement, Human and Health Sciences, Università degli Studi di Roma “Foro Italico”, Piazza Lauro De Bosis 6, 00135 Rome, Italy; 8 Center for Research in Neurobiology ‘Daniel Bovet’ (CRiN), Sapienza University of Rome, P.le Aldo Moro 5, 00185 Rome, Italy; 9 Institute of Biomembranes, Bioenergetics and Molecular Biotechnologies, National Research Council, 70126 Bari, Italy

**Keywords:** amyotrophic lateral sclerosis, spermidine, polyamines

## Abstract

Amyotrophic lateral sclerosis (ALS) is a fatal neurodegenerative disease marked by progressive degeneration of motor neurons and skeletal muscle. Gene expression analysis of the spinal cord and gastrocnemius of the SOD1-G93A ALS mouse model revealed a strong increase in inflammatory pathways and, specifically in the ALS gastrocnemius, a decrease in mitochondrial transcription and an increase in ribosomal protein expression. Treatment of ALS mice with the polyamine spermidine (SPD), a promising molecule in combating neurodegeneration and muscle atrophy, is able to partially restore the expression of more than four thousand genes in gastrocnemius tissue, including the mitochondrial regulator Pgc1α, as well as all the mitochondrial encoded genes and a large class of ribosomal proteins. SPD enhanced mitochondrial bioenergetics, as evidenced by Seahorse experiments, and delayed muscle weakness in vivo, as shown by grip strength records. These findings suggest that SPD can act as a potential supplement in the therapeutic strategy for ALS, offering a foundation for further research to improve patient outcomes.

## 1. Introduction

Amyotrophic lateral sclerosis (ALS) is a neurodegenerative disease with an incidence of 2 per 100,000 person-years. It is characterized by the degeneration of upper and lower motor neurons, leading to progressive voluntary muscle denervation; individuals may gradually lose the ability to speak, eat, move, and breathe [1]. Approximately 90–95% of cases are sporadic, while the remaining 5–10% are familial (fALS) and have different genetic mutations contributing to the disease. Several genes have been identified as causative of ALS, such as superoxide dismutase 1 (SOD1), TAR DNA-binding protein 43 (TDP-43), fused in sarcoma (FUS), and intronic hexanucleotide expansion (GGGGCC) in the C9orf72 gene.

Among the distinctive features of ALS, mitochondrial dysfunction [2] and neuroinflammation stand out [3]. All these aspects are extensively described in the SOD1-G93A mouse model, which recapitulates the pathology’s development in humans [4]. Traditionally, ALS research has focused primarily on the nervous system, with skeletal muscle degeneration considered a downstream effect of motor neuron loss. However, emerging evidence indicates that muscle dysfunction contributes to increased motor neuron susceptibility, particularly through metabolic dysregulation and oxidative stress. As a result, skeletal muscle is gaining recognition as a key therapeutic target in ALS [5]. Polyamines exert diverse pleiotropic effects by interacting with DNA, RNA, and proteins, stabilizing their structures and regulating gene expression [6,7,8]. Among PAs, spermidine (SPD) has been found to be an autophagy-inducer with positive effects on aging, tumorigenesis, and muscle-related diseases [9,10,11,12]. Previous studies have demonstrated that SPD mitigates rotenone-induced excitotoxicity in rats, leading to increased body weight and improved locomotor activity [13]. Furthermore, SPD possesses antioxidant properties, acting as a free radical scavenger to counteract oxidative stress in both neuronal and muscle cells [14]. It also plays a crucial role in modulating neuroinflammatory responses [15], promoting synaptic plasticity and neurogenesis [16].

Exogenous administration of SPD has been reported to ameliorate ALS phenotypes in SOD1-G93A mice. In particular, SPD treatment improved motor performance, delayed disease onset and extended survival. Nevertheless, at a molecular level, the effects of SPD supplementation have been investigated only in the spinal cord [17].

Considering these findings, we performed a comprehensive transcriptomic analysis of the spinal cord and skeletal muscle from SPD-treated SOD1-G93A mice to provide critical insights into the molecular mechanisms underlying the beneficial effects of SPD. Such an approach would help clarify its therapeutic potential, elucidate its impact on muscle-specific gene expression, and help to explain why SPD has been shown to prevent muscle atrophy and neurodegeneration [9,18,19].

## 2. Materials and Methods

### 2.1. Animals and Genotyping

All animal procedures were carried out in accordance with the European Guidelines for the use of animals in research (2010/63/EU) and the requirements of Italian law (D.L. 26/2014). All procedures were approved by the Animal Welfare Office, Department of Public Health and Veterinary, Nutrition and Food Safety, General Management of Animal Care and Veterinary Drugs of the Italian Ministry of Health (protocol number 293/2021-PR). Animals were kept in a virus/antigen-free facility with a light/dark cycle of 12 h at constant temperature and humidity. Food and water were provided ad libitum. SOD1-G93A mice (B6.Cg-Tg(SOD1 G93A)1Gur/J) were obtained from the Jackson Laboratory (Bar Harbor, ME, USA). Transgenic hemizygous SOD1-G93A males were crossbred with C57BL/6 females, and transgenic progeny were genotyped by PCR through hSOD1 oligos using interleukin-2 (IL-2) as a PCR internal control (Supplementary Table S2). Disease onset was evaluated by the hanging grid test; tests were conducted once per week starting at 55 days of age. To follow disease progression, behavioral scores and body weight were monitored starting at 55 days [20]. In brief, we assigned the following age (in days) to the following disease stages: 70 days as “symptoms onset”, 120 days as “symptomatic”, 150 days as “end stage”. Mice were anesthetized with Rompum (xylazine, 20 mg/mL, 0.5 mL/kg Bayer, Milan, Italy) plus Zoletil (tiletamine and zolazepam, 100 mg/mL, 0.5 mL/kg; Virbac, Milan, Italy) and then sacrificed for GNM and SC collection. We decided to collect the entire SC to eliminate technical variance associated with manual dissection boundaries, ensuring high sampling consistency and also enabling the identification of transcriptional features (e.g., neuroinflammatory response characterized by immune infiltration, microglial activation and astrogliosis) spread over the entire tissue. Only female mice were used in this study, in order to extend previous findings obtained from male SOD1-G93A mice [17] and to evaluate whether the effects of spermidine (SPD) are consistent across sexes.

### 2.2. Treatment with Spermidine and Evaluation of the Disease Progression

Spermidine (SPD) (S2501-25g, Sigma Aldrich (Merck), Darmstadt, Germany) was dissolved in drinking water at a concentration of 3 mM according to Eisenberg et al. [21] and administered to SOD1-G93A mice from the onset (70-day-old mice) to the symptomatic stage of the pathology (120-day-old mice). Control mice were given pure drinking water. Drinking water ± SPD was replaced every 2–3 days. Body weight and grip strength were measured once a week from the start until the end of the SPD treatment. For the grip strength test and body weight, we used: WT: *n* = 9, ALS: *n* = 8, ALS-SPD: *n* = 9. The grip strength test consisted of three attempts per mouse with a resting time of 1 min between every attempt; the mean of the three examinations, representing the strength of four limbs, was normalized to the weight of each respective mouse. The test was carried out by the same operator to minimize experimental variability.

### 2.3. RNA Isolation, Reverse Transcription and Real-Time PCR

Total RNA was extracted from the gastrocnemius (GNM) muscle and spinal cord (SC) of WT, SOD1-G93A-treated and SOD1-G93A-untreated mice (*n* ≥ 3) using TRIzol^®^ Reagent (Invitrogen, Carlsbad, CA, USA, 15596-018) and reverse-transcribed into cDNA using iScript Adv cDNA kit for RT-qPCR (Biorad, Hercules, CA, USA. #1725036) according to the manufacturer’s instructions. RNA was quantitatively and qualitatively evaluated using NanoDrop 2000c (ThermoFisher Scientific, Waltham, MA, USA) and Agilent Bioanalyzer 2100 (Agilent, Santa Clara, CA, USA), respectively. Levels of mRNA expression were measured by real-time quantitative PCR (qPCR) using the AriaMx Real-Time PCR System (Agilent Technologies, Santa Clara, CA, USA). PCR product quantification was calculated by applying the SYBR-Green (Bio-Rad Laboratories, Tokyo, Japan) method. The data are calculated relative to the internal housekeeping gene according to the second derivative test (2^−ΔΔCT^) method. Beta-2-microglobulin (B2m) was used as the housekeeping gene for normalization in GNM, while glyceraldehyde-3-phosphate dehydrogenase (Gapdh) was used as the housekeeping gene for normalization in SC. Primers sequences are listed in Supplementary Table S3.

### 2.4. RNA Library Construction and Sequencing

Total RNA was subjected to RNA-seq at the CNR BIOMICS facility of the Italian node of the European Research Infrastructure ELIXIR (CNR-IBIOM, Bari, Italy). Four biological replicates for GNM and three biological replicates for SC from each biological condition were analyzed. RNA-seq libraries were prepared from 300 ng of total RNA, using the Illumina Stranded Total RNA Prep with Ribo-Zero Plus (Illumina, San Diego, CA, USA), according to the manufacturer’s protocol. cDNA libraries were checked with Bioanalyzer 2100 and quantified by fluorimetry using the Qubit dsDNA Assay Kit (Thermo Fisher Scientific). Sequencing was performed on the Illumina Novaseq 6000 platform, generating 100pb paired-end reads. All computations were performed on machines running GNU + Linux (3.10.0–862.14.4.el7.x86_64) by using R (version 3.6.1) and Bash (4.2.46(2)-release x86_64-redhat-linux-gnu). The quality of the RNA-seq reads was preliminarily inspected with fastQC [22] and MultiQC [23], and no trimming was performed since sequencing adapters were removed during the demultiplexing step with bcl2fastq (version 2.20) (https://support.illumina.com/sequencing/sequencing_software/bcl2fastq-conversion-software.html, accessed on 15 March 2026), and the reads’ mean quality score per base was greater than 25 for all samples. All steps of the analysis, dependent on genomic and transcriptomic annotation, were performed employing version 31 of GENCODE’s murine GTF and FASTA files (https://www.gencodegenes.org/mouse/release_M31.html, accessed on 15 March 2026). Reads were aligned with the GRCm39 murine genome by means of STAR [24] (version 2.5.2b), and by using the --outFilterMultimapNmax 1 option, all multimapping reads were discarded. The summarization of paired-end fragments to genes was performed with FeatureCounts [25] (version 1.6.0). The process of read alignment with the reference mouse genome uniquely mapped (i) 81.7% of the ∼167 million input reads, namely ∼11 million reads on average per sample for GNM and (ii) 90.3% of the ∼543 million input reads, namely ∼41 million reads on average per sample for SC. Multidimensional Scaling (MDS) analyses were performed to study data structure, identify potential outliers and measure the total variability among samples, identifying clusters based on genic expression patterns. MDS analyses were carried out with the regularized log-transformed gene counts (obtained with the DESeq2 rlog function) and the cmdscale R function; in particular, the Euclidean distance was adopted for the MDS by using the dedicated option of cmdscale. Heatmaps were performed by hierarchical clustering of DESeq2 normalized counts using the ggplot R package (version 3.1.3.1).

Data were prepared for the differential expression analysis as necessary via a custom R script. DESeq2 [26] (version 1.26.0) was used to perform the normalization of raw sequencing counts with DESeq2’s mean log ratio method and the differential expression analysis between the experimental conditions of interest. Before extracting the results of the differential expression analysis, a preliminary gene expression filter was employed, and genes whose sum of normalized counts was less than 10 in half the samples of the dataset were discarded to focus only on highly expressed genes. Q-values were computed [27] with the qvalue R package, and genes were considered differentially expressed when presenting a q-value ≤ 0.05.

### 2.5. Mitochondrial DNA/Nuclear DNA Ratio Evaluation

DNA was extracted from gastrocnemius tissue using 450 μL of lysis buffer solution composed of 445.5 μL of lysis buffer (Tris-HCl 1 M pH 8; EDTA 0.5 M pH 8; NaCl 5 M; SDS 10%) and 4.5 μL of proteinase K (10 mg/mL) to degrade the proteins present in the tissue sample. Samples were incubated O/N at 56 °C. RNase A (100 µg/mL) was added to degrade the RNA present through incubation at 37 °C for 30 min. Samples were then centrifuged for 10 min at 16,100× *g*, and NaCl 5M was added to the supernatant. After 10 min on a rocking platform, the samples were centrifuged again for 10 min at 16,100× *g*. Cold isopropanol was added to the supernatant and centrifuged for 10 min at 16,100× *g* at 4 °C. The pellet containing DNA was washed with ethanol 80% and suspended in 20 μL of double-distilled water (Milli-Q^®^ SQ 200P). Different genes were selected to evaluate the relative copy number of mtDNA and nDNA by qPCR. In the mouse mitochondrial genome, genes corresponding to the stable fraction that is not prone to deletions encode for mt-Rnr2 (16S rRNA). We used mt-Rnr2 and mt-Co1 for mt-encoded genes and B2m and Ndufv1 as nuclear genes. Calculation of the mtDNA/nDNA ratio was performed using the ΔΔCT method. Primers are listed in Supplementary Table S4.

### 2.6. Gene Set Enrichment (GSEA) Analysis of Differentially Expressed Genes

We used the WebGestAlt R package [28,29] (version 0.4.6) to perform GSEA analysis of ALS vs. CTR and ALS + SPD vs. ALS comparison. The WikiPathway database was used to determine up- or down-regulated pathways. The following settings were used: FDR ≤ 0.05, minimum number of genes per set = 5, maximum number of genes per set = 2000, permutation number = 10.000, gseaP = 0. Barplots were made in R using ggplot2 (version 3.5.1)

### 2.7. Bioenergetic Analysis

The bioenergetic analysis was performed with the Seahorse XF96e Analyzer (Seahorse Bioscience—Agilent, Santa Clara, CA, USA), through the Cell Mito Stress Test. C2C12 cells were transfected with CMV plasmid coding for human wild-type SOD1 and human SOD1 with the mutation G93A seeded at a density of 8 × 10^3^ live cells per well on Seahorse XF tissue-culture-treated microplates (Agilent Technologies, Santa Clara, CA, USA, cat. no. 103794-100). The cells were treated O/N with different concentrations of SPD (1 μM, 10 μM, 30 μM), dissolved in the culture medium before the experiment. Doxycycline-inducible NSC34 cells stably expressing 5 × Myc-tagged human mutant Q331K TDP-43, previously generated in our laboratory [30], were pre-treated with doxycycline (1 µg/mL) for 48 h. Subsequently, 20 × 10^3^ NSC34 cells were seeded on micro-lysine-coated XF96 microplates and O/N treated exclusively with the selected SPD concentration (1 µM), always in the presence of doxycycline.

Briefly, for the Cell Mito Stress Test, the growth medium was replaced with Seahorse XF Dulbecco’s Modified Eagle Medium, pH 7.4 (Seahorse Bioscience—Agilent, Santa Clara, CA, USA), supplemented with 1 mM sodium pyruvate, 10 mM glucose, and 2 mM L-glutamine. The cells were incubated at 37 °C without CO_2_ for 45 min before the assay to allow for equilibration with the assay medium.

The Cell Mito Stress Test was conducted according to the manufacturer’s instructions to evaluate mitochondrial function by monitoring the oxygen consumption rate (OCR) in real time. Various inhibitors were sequentially injected to perturb the electron transport chain complexes: oligomycin (1.5 µM), an ATP synthase inhibitor, to determine ATP production-coupled respiration; carbonyl cyanide-p-trifluoromethoxyphenylhydrazone (FCCP) (1 µM), an uncoupler, to measure the maximal respiration rate; and a combination of rotenone (0.5 µM) and antimycin A (0.5 µM), inhibitors of complexes I and III respectively, to assess non-mitochondrial respiration.

The assay enabled the determination of key parameters of mitochondrial function, including basal respiration (baseline OCR before oligomycin addition), ATP-linked respiration (difference between basal respiration and the minimal respiration after oligomycin), maximal respiration (OCR following FCCP addition), and spare respiratory capacity (difference between maximal respiration and basal respiration, indicating the cell’s ability to respond to a greater demand for energy or under stress). After the assay, cells were lysed with 10 µL of 0.1% SDS in water, and protein quantitation was performed to normalize OCR data, which were expressed as pmol O_2_/min/mg of protein. The data were analyzed using the Seahorse Analytics online platform (Agilent Technologies, https://seahorseanalytics.agilent.com/, accessed on 15 March 2026) or the XFe Wave software (V2.6.4.24) (Santa Clara, CA, USA).

### 2.8. Statistical Analysis

The results are presented as means ± SEM of *n* ≥ 3 independent experiments. For the analysis of weight, grip strength tests, RNA expression and polyamine concentration, data were evaluated using one- or two-way ANOVA as required, with the Tukey test as a post hoc analysis. Differences between groups were considered significant when the *p*-value was less than *p* < 0.05. The normality of the data was assessed using QQPlot and the Shapiro–Wilk test. Correlation analyses within RNA-seq and qPCR were assessed using Spearman correlation with a two-tailed *p*-value. Significant correlations between DESeq2 count and 2^−ΔCT^ were considered significant when the *p*-value was less than *p* < 0.05. Statistical analysis was performed using GraphPad Prism 8.0.1.

## 3. Results

### 3.1. RNA-Seq Revealed Both Distinct and Common Pathways in SOD1-G93A Spinal Cord and Skeletal Muscle Tissues

**Figure 1:**
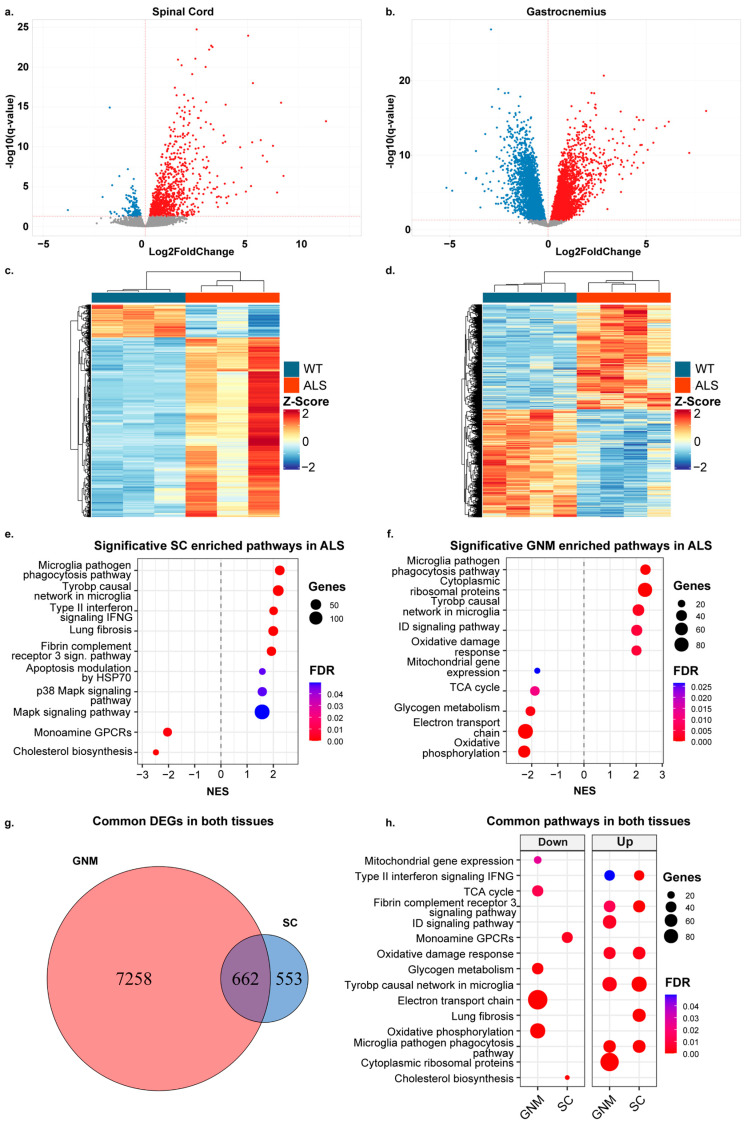
Transcriptomic analysis revealed deregulated genes and pathways in spinal cord and skeletal muscle of SOD1-G93A mice. (**a**,**b**) Volcano plot of differential gene expression in SOD1-G93A mice versus control mice using the Wald test for spinal cord (SC) and gastrocnemius (GNM) tissues. The statistically significant up-regulated and down-regulated genes are shown in red and blue respectively. (**c**,**d**) Heatmap of differentially expressed genes in spinal cord and gastrocnemius, respectively, between SOD1-G93A and control mice. Each row of the heatmap represents the z-score-transformed DESeq2 values of one differentially expressed gene across all samples. The row z-score is represented by a color code (blue, down-regulated genes; red, up-regulated genes). (**e**,**f**) Dotplot of significant pathways derived from gene set enrichment analysis (GSEA) for SC and GNM tissue. The colors represent the *p*-value adjusted by Benjamini–Hochberg correction. The dot dimension represents the number of DEGs for that pathway. NES, normalized enrichment score. (**g**) Venn diagram showing the number of differentially expressed genes (DEGs) in GNM and SC. Common DEGs are indicated in the intersection. (**h**) Comparative dotplot of common deregulated pathways between tissues.

**Figure 2:**
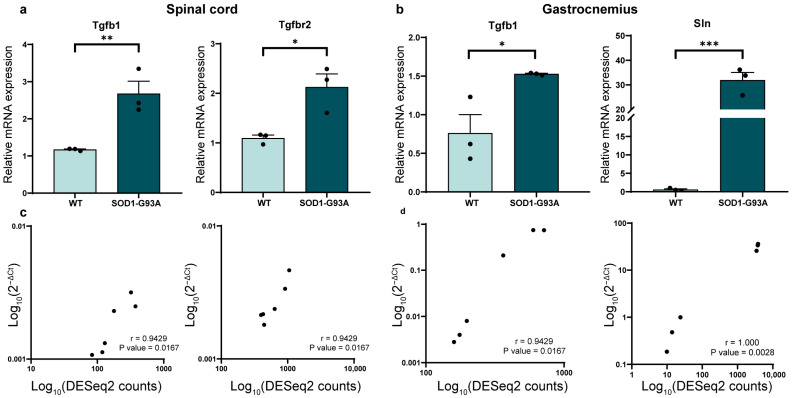
Validation of RNA-seq analysis in spinal cord and gastrocnemius of SOD1-G93A mice. (**a**,**b**) RT-qPCR on two deregulated genes from spinal cord (SC) and gastrocnemius (GNM) RNA-seq analysis. Data are represented as mean ± SEM with 2^−ΔΔCt^ method for *n* = 3 mice per experimental group. Data were normalized using Gapdh and beta-2-microglobulin (B2m) as housekeeping genes for SC and GNM respectively. Unpaired *t*-tests were used; * *p* < 0.05, ** *p* < 0.01, *** *p* < 0.001. (**c**,**d**) Spearman’s correlation test between 2^−ΔCt^ values and DESeq2 counts from RT-qPCR and RNA-seq analysis, respectively. *p*-value and Spearman’s r coefficient are displayed in the graphs.

Our first goal was to better understand the transcriptomic profile of ALS mice in the two main tissues affected by the pathology. To identify differentially expressed genes, we analyzed spinal cord (SC) and gastrocnemius (GNM) tissues dissected from wild-type (WT) mice (control group) and SOD1-G93A 120-day-old mice (ALS group), a stage corresponding to the symptomatic phase of the ALS model. We isolated RNA from SC and GNM and performed bulk RNA sequencing (RNA-seq) analysis. Multidimensional Scaling (MDS) (Supplementary Figure S1) showed a clear separation of the ALS group compared to the control group for both tissues. Differentially expressed gene (DEG) analysis identified several transcripts deregulated in ALS (Supplementary Data S1 and S2): a total of 1215 genes deregulated for SC, with 1028 up-regulated and 187 down-regulated, and 7929 deregulated genes in GNM, with 3906 up-regulated and 4023 down-regulated (Figure 1a,b and Supplementary Table S1). A heatmap of the DEGs showed significant alterations in the ALS expression profiles in both tissues (Figure 1c,d). We compared our results with the ALSoD (https://alsod.ac.uk/, accessed on 15 March 2026) database, which lists genes associated with ALS. Among the 154 ALS-associated genes, we found 17 DEGs (11.04%) in SC and 82 DEGs (53.25%) in GNM of our samples. In particular, in SC, 12 genes were up-regulated (including Apoe and Cx3cr1), and five were down-regulated (Nefl and Nefh); in GNM, 45 genes were up-regulated (including SOD1, Apoe, Anxa11, and Fggy), and 37 were down-regulated (including Optn, Taf15, and Vapb) (Supplementary Data S3 and S4). Gene set enrichment analysis (GSEA) identified significant pathways (FDR ≤ 0.05) associated with ALS in SC and GNM. In SC, enrichment analysis revealed an increased expression of inflammation-related pathways (e.g., TYROBP causal network, Type II interferon, and the microglia pathogen phagocytosis pathway) and reduced expression in Cholesterol Biosynthesis and monoamine receptors (Figure 1e). In GNM, the analysis showed an up-regulation of some inflammatory pathways (e.g., TYROBP causal network) and Cytoplasmic Ribosomal Proteins. Down-regulated pathways included glycogen metabolism and mitochondria-related pathways (oxidative phosphorylation and the electron transport Chain) (Figure 1f and Supplementary Figure S1a). Moreover, we compared DEGs from SC and GNM, finding a total of 662 common DEGs between them (Figure 1g). The enrichment analysis of these common DEGs showed that oxidative damage and inflammation are key pathways deregulated in both tissues (Figure 1h). For SC, we validated the expression profile of two markers of astrogliosis, Tgfb1 and Tgfbr2 (Figure 2a), that appeared up-regulated in ALS mice, thus confirming an aberrant and reactive profile of astrocytes surrounding motor neurons [31]. Tgfb1 was also up-regulated in GNM from ALS mice (Figure 2b), consistent with its inhibition of myoblast differentiation [32]. ALS skeletal muscle is characterized not only by inflammation but also by a dysregulated metabolism [33,34], and, in line with this, in ALS mice, we found increased expression of Sln, a marker in the transition toward oxidative metabolism [35] (Figure 2b). Spearman’s correlation confirmed a strong, significant correlation between DESeq2 counts and qPCR expression levels.

### 3.2. Spermidine Treatment Modifies the Transcriptomic Profile in SOD1-G93A Mouse Model

**Figure 3:**
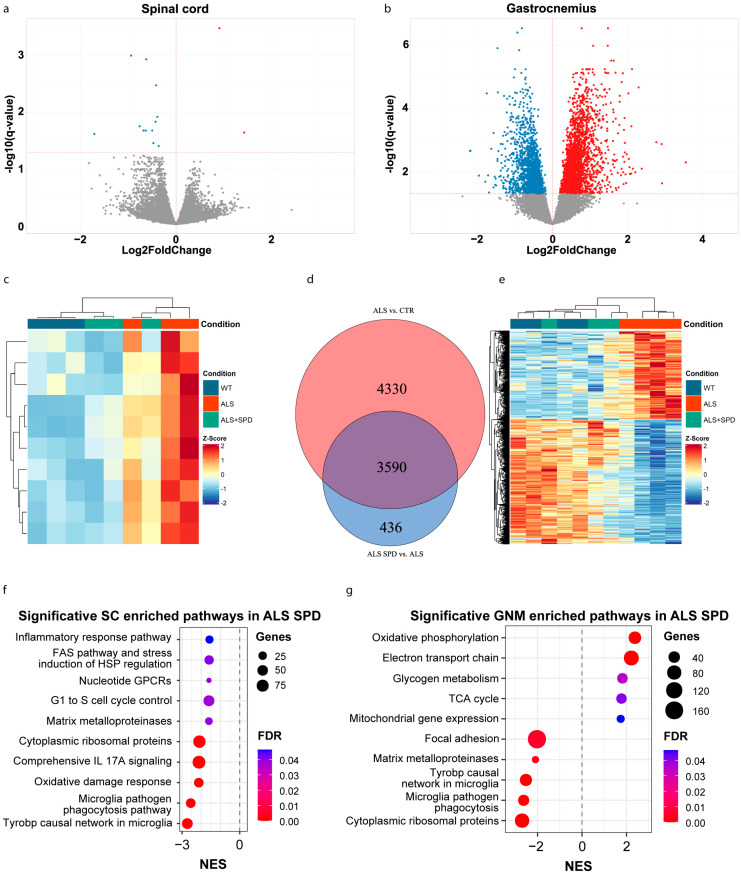
Transcriptomic analysis of spinal cord and skeletal muscle of SOD1-G93A and SOD1-G93A treated with SPD. (**a**,**b**) Volcano plot of differentially expressed genes from spinal cord (SC) and gastrocnemius (GNM) tissues of SOD1-G93A and SOD1-G93A mice treated with SPD (SOD1-G93A SPD). The statistically significant up-regulated and down-regulated genes are shown in red and blue respectively, while grey points represent not significant genes. (**c**) Heatmap of the 10 genes whose expression is reverted in the WT vs. ALS and ALS vs. ALS + SPD comparisons for SC. (**d**,**e**) Venn diagram and heatmap of 3590 common genes between WT vs. ALS and ALS vs. ALS + SPD for GNM. In Venn diagram are represented DEGs in a red circle for ALS vs. CTR comparison, while in a blue circle for ALS SPD vs. ALS comparison. Each row of the heatmap represents the z-score-transformed DESeq2 values of one differentially expressed gene (DEG) across all samples. The row z-score is represented by a color code (blue, down-regulated genes; red, up-regulated genes). (**f**,**g**) Dotplot of significant pathways derived from gene set enrichment analysis (GSEA) for SC and GNM tissue respectively. The colors represent the *p*-value adjusted by Benjamini–Hochberg correction. The dot dimension represents the number of DEGs for that pathway. NES, normalized enrichment score.

Due to the well-known neuroprotective, anti-inflammatory and antioxidant roles of exogenous SPD and its beneficial effects previously reported in ALS [17], we decided to evaluate the expression profiles after SPD treatment in both GNM and SC tissues (referred to as the ALS_SPD group). In ALS_SPD SC, we detected 14 deregulated genes, one lncRNA and 13 protein-coding genes (Supplementary Data S5). As shown in the volcano plot, most of these genes were down-regulated, with only two genes showing up-regulation (Figure 3a,e). Interestingly, the heatmap showed clustering of three ALS_SPD mice with the WT mice cluster (Figure 3c). From the RNA-seq analysis conducted on the GNM of ALS and ALS_SPD mice, we found 4028 deregulated genes (Figure 3b) (Supplementary Data S6), 3590 of which were in common with the ALS vs. CTR comparison, as shown in the Venn diagram (Figure 3d). The heatmap (Figure 3d) showed a clustering of two out of three mice treated with SPD closer to the WT mice, while the ALS_SPD2 sample appears to be like the ALS samples. We then compared genes whose expressions were modified by the SPD treatment with those in the ALSoD database and found that 39 genes (27.92%) were common. Among these, 27 genes were up-regulated (including Matr3, Atxn1, and Odr4), and 12 genes were down-regulated (including Sqstm1, Apoe, and Cst3). Of these 39 genes, 36 had reverted expression compared to the ALS vs. CTR comparison (Supplementary Data S4). Among the down-regulated pathways, enrichment analysis identified inflammation-related pathways (such as the inflammatory response pathway and microglia pathogen phagocytosis pathway) and the Cytoplasmic Ribosomal protein in SC (Figure 3f); among the down-regulated pathways, it identified inflammation-related pathways (such as the Tyrobp causal network in microglia and the microglia pathogen phagocytosis pathway) and Cytoplasmic Ribosomal protein in the GNM; and among the up-regulated pathways, it identified oxidative phosphorylation and the electron transport chain (Figure 3g and Supplementary Figure S3b). Since mitochondrial dysregulation is a hallmark of ALS, the partially normalized expression of mitochondria-related pathways in the ALS_SPD samples suggests an effect of SPD on cellular energy machinery.

**Figure 4:**
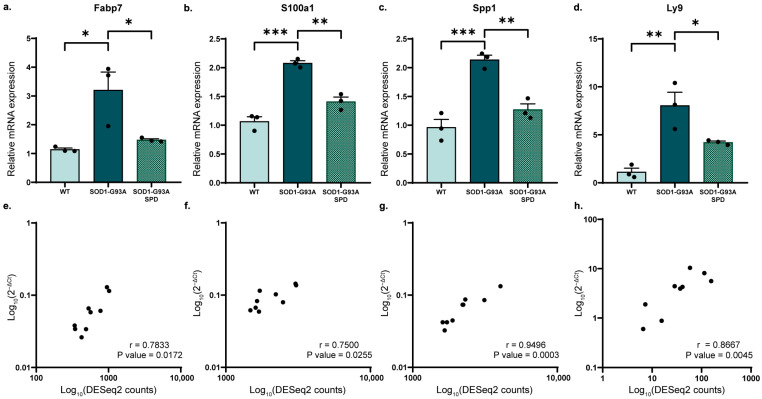
Validation of some DEGs in spinal cord tissue of SOD1-G93A mice after SPD treatment. (**a**–**d**) RT-qPCR of deregulated genes from RNA-seq analysis for spinal cord. Data are represented as mean ± SEM using expression levels measured using 2^−ΔΔCt^ method for *n* = 3 mice per experimental group. Data were normalized using Gapdh as housekeeping gene. One-way ANOVA, followed by Tukey’s post hoc test, was used; * *p* < 0.05, ** *p* < 0.01, *** *p* < 0.001. (**e**–**h**) Spearman’s correlation test was used to assess a correlation between 2^−ΔCt^ values and DESeq2 counts from RNA-seq analysis. *p*-value and Spearman’s r coefficient are displayed in the graphs.

In SC, we validated Fabp7, S100a1, Spp1 and Ly9. All these genes have a role in the inflammatory response. All of them were up-regulated in ALS and recovered by the SPD treatment (Figure 4a–d). Spearman’s correlation analysis confirmed a strong, significant correlation between DESeq2 counts and qPCR fold change (Figure 4e–h).

**Figure 5:**
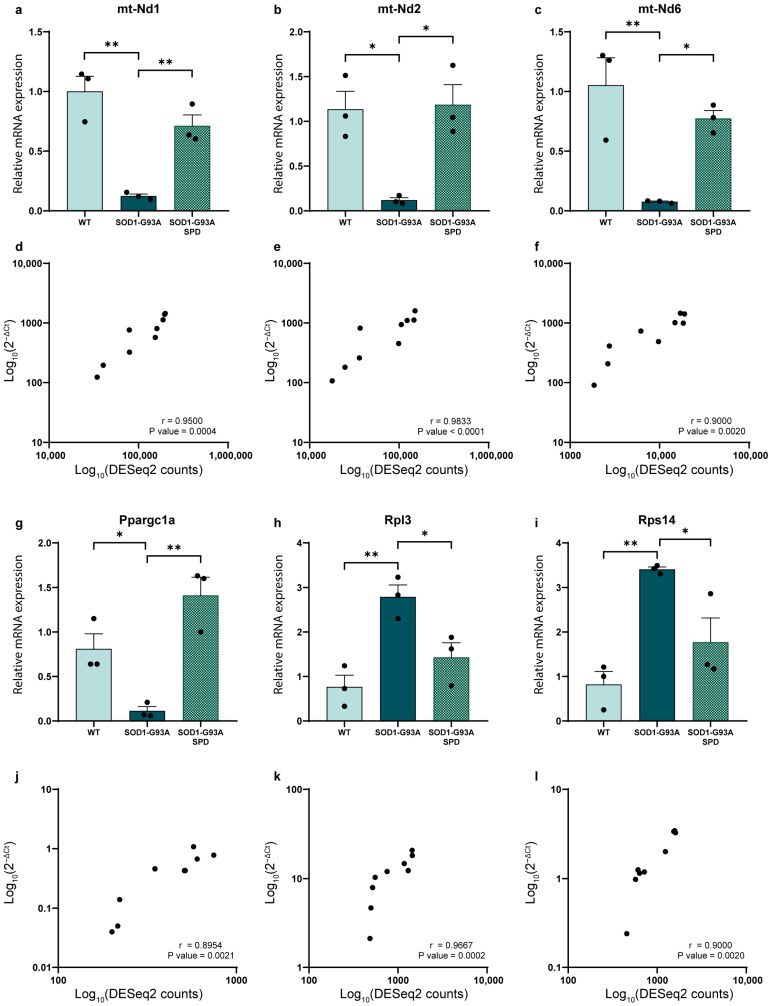
Validation of genes related to mitochondrial and ribosomal protein pathways emerging from GSEA in gastrocnemius tissue of SOD1-G93A mice after SPD treatment. (**a**–**c**,**g**–**i**) RT-qPCR of deregulated genes from RNA-seq analysis of gastrocnemius. Data are mean ± SEM represented using expression levels measured using 2^−ΔΔCt^ method for *n* = 3 mice per experimental group. Data were normalized using beta-2-microglobulin (B2m) as housekeeping gene. One-way ANOVA, followed by Tukey’s post hoc test, was used; * *p* < 0.05, ** *p* < 0.01. (**d**–**f**,**j**–**l**) Spearman’s correlation test was used to assess a correlation between 2^−ΔCt^ values and DESeq2 counts from RNA-seq analysis, *n* = 3 mice per experimental group. *p*-value and Spearman’s r coefficient are displayed in the graphs.

In GNM, we validated genes from the two most enriched pathways: Oxidative Phosphorylation and electron transport chain (up-regulated) and Cytoplasmic Ribosomal Proteins (down-regulated). For the mitochondria-related pathway, we assessed the expression of mt-Nd1, mt-Nd2 and mt-Nd6, showing that their expression levels decreased in ALS and were attenuated by the SPD treatment (Figure 5a–c). Additionally, we assessed the expression level of Ppargc1a, which codifies Pgc1α, a regulator of mitochondrial biogenesis and mtDNA transcription. Confirming the RNA-seq data, Pgc1α expression is decreased in ALS and rescued by SPD administration (Figure 5g). Spearman’s correlation analysis confirmed a strong, significant correlation between DESeq2 counts and qPCR fold change (Figure 5d–f,j–l). Mitochondrial deregulation has been widely described in ALS pathology [36,37,38], and accordingly, we found that all mt-DNA-encoded genes were deregulated. Interestingly, SPD treatment seems to revert this dysregulation (Table 1).

To ensure that the effect of SPD was not at the mtDNA level, we analyzed the mtDNA/nDNA ratio, and we found no significant differences among all groups compared to WT control mice (Supplementary Figure S2). For the Cytoplasmic Ribosomal Proteins pathway, we validated the expression of Rpl3 and Rps14 through qPCR (Figure 5h,i). Both genes showed increased expression levels in the pathological condition, while their expression was significantly reduced following SPD treatment, suggesting a potential modulatory effect of SPD on ribosomal protein dysregulation.

Collectively, these results highlight the profound positive effects of SPD treatment on both SC and GNM tissues.

### 3.3. Spermidine Treatment Improves Mitochondrial Metabolism in ALS Cellular Model and Muscle Force in ALS Mouse Model

**Figure 6:**
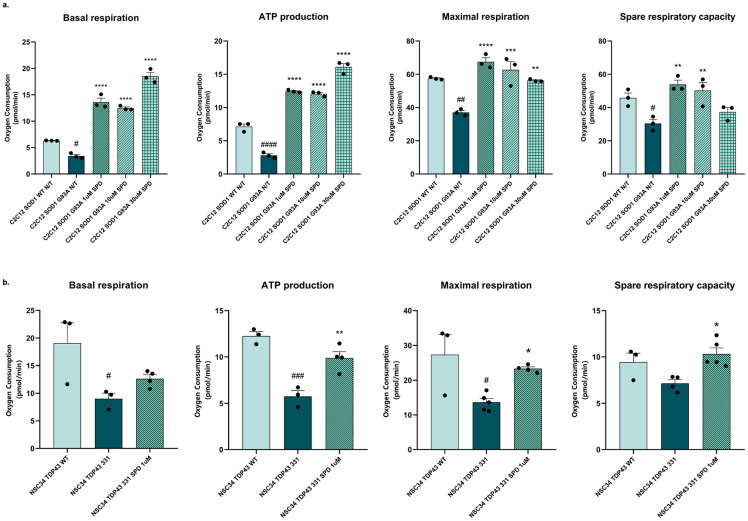
Spermidine has a positive effect on mitochondrial bioenergetics in C2C12 cells expressing hSOD1-G93A mutation (**a**) and in NSC34 expressing hTDP43 Q331K mutation (**b**). Individual parameters for basal respiration, maximal respiration, ATP production and spare respiratory capacity were measured. Data are shown as mean ± SEM. One-way ANOVA, with Tukey’s post hoc test, was used. Values significantly different from control (WT) are indicated with # *p* < 0.05, ## *p* < 0.01, ### *p* < 0.001, #### *p* < 0.0001. Values significantly different from ALS condition (SOD1 G93A and TDP43 331) are represented with * *p* < 0.05, ** *p* < 0.01, *** *p* < 0.001, **** *p* < 0.0001.

Given the crucial role of mitochondrial metabolism in cellular energy homeostasis and the marked dysregulation that occurs in the skeletal muscle of SOD1-G93A mice, we assessed the bioenergetic profile of SPD-treated and untreated C2C12 myoblast cell lines transiently transfected with the human form of SOD1 (hSOD1) carrying the G93A mutation, as well as C2C12 transfected with the wild-type (WT) hSOD1 protein. An analysis of the oxygen consumption rate (OCR) revealed a significant decrease in basal respiration, maximal respiration, ATP production and spare respiratory capacity in C2C12 SOD1-G93A cells compared to the control, while SPD administration, even at the lowest concentration (1 μM), is effective in recovering the above-mentioned parameters (Figure 6).

We conducted the same analysis using the motor-neuron-like NSC34 cell line carrying the ALS-related TDP-43 Q331K mutation. Given that the concentrations of SPD used in the C2C12 assay did not produce a significant difference in treatment effects, we selected the lower effective concentration (1 µM). In NSC34 TDP-43 Q331K cells, OCR analysis revealed dysregulation in basal respiration, maximal respiration, and ATP production, all of which were counteracted by the SPD treatment (Figure 6b), further supporting its beneficial effect in a neuronal context. These results highlight improved mitochondrial bioenergetics in ALS muscle and motor neuron cell lines after SPD treatment.

**Figure 7:**
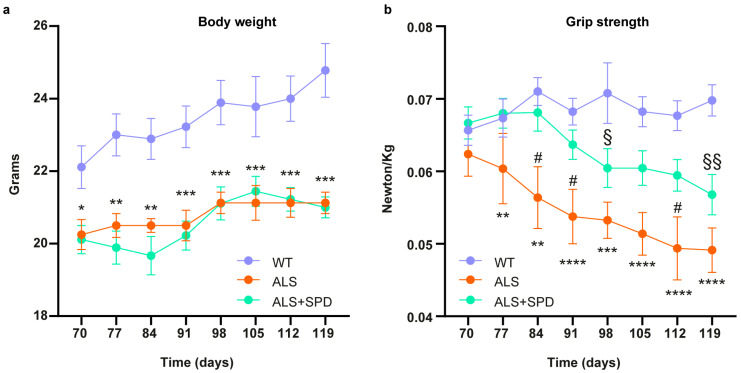
Body weight and grip strength analyses of SOD1-G93A mice treated with SPD. (**a**) Graph representing the weight of WT, SOD1-G93A and SOD1-G93A + SPD mice at the indicated ages. Data are presented as the means ± SEM; * *p* < 0.05, ** *p* < 0.01, *** *p* < 0.001 for ALS vs. WT comparison. (**b**) Maximal grip strength for WT, SOD1-G93A and SOD1-G93A + SPD mice. Data are presented as the means ± SEM; ** *p* < 0.01, *** *p* < 0.001 and **** *p* < 0.0001 for ALS vs. WT comparison; # *p* < 0.05 for ALS vs. ALS + SPD comparison; § *p* < 0.05 and §§ *p* < 0.01 for ALS SPD vs. WT comparison. Two-way ANOVA Multiple comparisons test was used.

Finally, we analyzed body weight and muscle strength in SPD-treated and control group mice. SOD1-G93A mice showed a lower body weight throughout the treatment period compared to wild-type (WT) mice, and the SPD treatment did not mitigate the weight loss associated with ALS (Figure 7a). However, a different scenario was observed for grip strength. While WT mice maintained consistent strength from the onset to the end of the evaluation, SOD1-G93A mice exhibited a significant decrease, as expected. Interestingly, SPD treatment delays the loss of muscle strength in ALS mice (Figure 7b). Collectively, our results showed that SPD treatment can ameliorate mitochondrial bioenergetics at the cellular level and delay muscle strength deterioration in the SOD1-G93A mouse model.

## 4. Discussion

In this study, we analyzed the transcriptomic profiles of ALS mice, with or without SPD treatment, focusing on two key tissues affected by the disease: SC and GNM. While previous studies have often restricted analysis to the lumbar region of the SC [39] to maximize sensitivity for motor-neuron-related changes, we opted to analyze the entire tissue, enabling the identification of shared and global transcriptional signatures (e.g., neuroinflammatory response characterized by immune infiltration, microglial activation, and astrogliosis) that may not be captured when focusing on a single segment [40,41]. Furthermore, the use of female mice in our experimental design was a deliberate choice aimed at complementing the existing literature. Since the beneficial effects of SPD have been previously demonstrated in male SOD1-G93A mice [17], our findings confirm that these neuroprotective outcomes are consistent across sexes, strengthening the generalizability of SPD efficacy. This is particularly relevant given the well-documented sexual dimorphism in the SOD1-G93A model, especially regarding pathways central to our study, such as mitochondrial function and neuroinflammation [42]. Indeed, female mice exhibit distinct mitochondrial stress responses [43,44], and sex hormones are known to modulate neuroinflammatory processes in the spinal cord [45]. Investigating female cohorts, therefore, provides biologically relevant and complementary insights into the mechanisms underlying ALS progression and SPD-mediated protection.

Regarding the most altered pathways in ALS, our findings, in line with other studies [46,47,48,49,50], show that inflammation plays a significant role in both the SC and GNM, as evidenced by the shared inflammatory gene profiles between these tissues (Figure 1h). Notably, Tgfb1, encoding a secreted ligand of the TGFb (transforming growth factor-beta) superfamily of proteins, a critical player in muscle regeneration [51], neuronal survival, development and maintenance [32], was highly expressed in both tissues. This elevated expression suggests the potential onset of reactive astrogliosis [52,53] and impaired muscle regeneration [54]. TGFb inhibits both muscle cell proliferation and differentiation and influences muscle fiber patterning during regeneration following injury. Additionally, TGFb family members are crucial for promoting fibrosis and scar tissue formation, as they enhance extracellular matrix (ECM) production [55]. In SOD1-G93A GNM from SPD-treated mice, we observed a reduction of Tgfb1 (Supplementary Data S4), suggesting a reduction in the progression of fibrosis [55].

While Tgfb1 expression was deregulated in both SC and GNM, Tgfbr2 expression was increased only in SC. Interestingly, elevated levels of Tgfbr2 in the SC have been linked to reactive astrogliosis and disease progression in ALS [32,56].

In SC, SPD treatment modulates the expression of 13 protein-coding genes, including Fabp7, which regulates lipid metabolism and inflammation [57]. Notably, Fabp7 was up-regulated in ALS but down-regulated following SPD treatment. Its up-regulation in astrocytes promotes a pro-inflammatory phenotype that can be toxic to motor neurons [58]. The up-regulation of Fabp7 also triggers pro-inflammatory responses in human astrocytes and has been observed in the brains of Alzheimer’s disease patients and mouse models, as well as in the SC of ALS mouse models [58,59]. Additionally, Spp1 has emerged as a neuroinflammation biomarker, playing a crucial role in immune cell recruitment and neuroglial interactions within the CNS [60]. Spp1 is particularly up-regulated in the SC microglia of ALS mice [60,61]. S100a1, which is involved in calcium homeostasis, has been linked to exacerbated neuroinflammation in other models [62], and Ly9 is involved in immune regulation [63]. The decrease after SPD treatment in the expression of these four key inflammatory markers may suggest a mechanism through which SPD reduces inflammation in the ALS SC.

**Figure 8:**
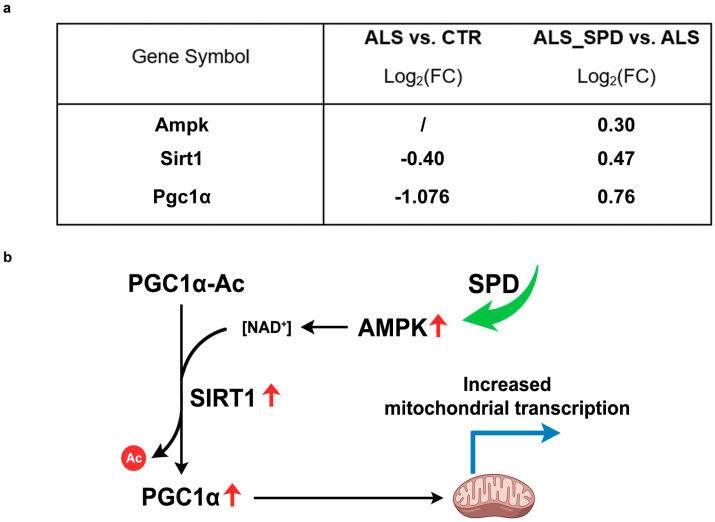
Proposed effect of SPD treatment on mitochondrial bioenergetics in GNM. (**a**) Table showing the Log2(FoldChange) for the ALS vs. CTR and ALS_SPD vs. ALS comparison of indicated genes. Data is extracted from Supplementary Data S2 and S6. (**b**) Schematic mechanism of SPD effect on GNM. Red arrows: transcriptional up-regulation; green arrow: SPD supplementation; Black arrows: enzymatic reaction and downstream signaling; blue arrow: increased transcription; AMPK: AMP-activated protein kinase, NAD^+^: nicotinamide adenine dinucleotide, PGC1α: peroxisome proliferator-activated receptor gamma coactivator 1-alpha, PGC1α-AC: acetylated PGC1α, SIRT1: Sirtuin 1.

In ALS GNM, our analysis revealed a global change in gene expression (almost 8000 DEGs). The mitochondrial DNA (mtDNA)-encoded genes were down-regulated, suggesting impaired mitochondrial transcription. This reduced transcriptional activity may compromise mitochondrial function, as observed by us and others in ALS cellular models [64,65,66,67,68], potentially contributing to muscle wasting. The observed down-regulation is likely associated with the decreased activity of mitochondrial-specific transcription factors. Consistent with previous studies [69,70], we identified a reduction in Pgc1α expression in ALS muscle, a key regulator of mitochondrial transcription [71,72]. Notably, the SPD treatment promoted a partial normalization of Pgc1α transcript levels. Based on this observation, we hypothesize that the modulation of this key regulator could represent one of the primary mechanisms through which SPD mitigates mitochondrial dysfunction [73] and attenuates the dysregulation of mtDNA-encoded genes (Figure 6 and Table 1). Moreover, AMP-activated protein kinase (AMPK), a kinase able to enhance Pgc1α expression and activity, was also found to be down-regulated in ALS and up-regulated after SPD treatment in our datasets (Figure 8a). Spermidine is known to induce autophagy and metabolic reprogramming primarily through the phosphorylation and activation of AMPK [10,21]. Activated AMPK canonically acts as an upstream regulator that directly phosphorylates Pgc1α and concomitantly activates SIRT1, which subsequently deacetylates Pgc1α to maximize its transcriptional activity [74]. Therefore, we hypothesize that the SPD-induced up-regulation of AMPK is a primary upstream event driving the amelioration in Pgc1α expression and subsequent mitochondrial rescue. To our knowledge, this is the first report of SPD-induced modulation of AMPK at the transcriptional level in this model. These findings suggest that SPD may exert its protective effects, at least in part, through modulation of the AMPK/Pgc1α signaling axis (Figure 8b), highlighting a mechanism that deserves further investigation.

RNA-seq analysis also revealed the increased expression of several RPs in the GNM of SOD1-G93A ALS mice, which were partially restored by the SPD treatment (Figure 5h,i). RPs are involved in the stabilization of the small and large subunits of ribosomes, in the regulation of cell growth, proliferation and differentiation, immune signaling, DNA repair and apoptosis [75]. Although the role of RPs in ALS is not well understood, some studies have noted their deregulation in induced pluripotent stem cells of C9ORF72 ALS patients [76,77,78]. Emerging evidence suggests that, beyond their canonical functions, many RPs play crucial roles in regulating physiological and pathological processes, including inflammation. While some RPs act as protective “gatekeepers,” others may function as “Trojan horses,” exacerbating inflammatory responses [79]. For the first time, RP dysregulation was found in the GNM of the SOD1-G93A mouse model, with the SPD treatment significantly attenuating these alterations. The precise cause-and-effect relationship by which SPD reduces RP expression requires further validation. However, SPD is the exclusive precursor for the hypusination of eukaryotic translation initiation factor 5A (eIF5A), which globally regulates ribosomal pausing and translation elongation [80]. In the context of ALS, motor units frequently suffer from endoplasmic reticulum (ER) stress due to proteotoxicity. So, the down-regulation of RPs observed in our dataset might represent an SPD-mediated compensatory mechanism, where the attenuation of global translation rates and ribosomal assembly driven by SPD may relieve ER stress and restore proteostasis [10,81]. Considering the well-established anti-inflammatory properties of SPD and its role in proteostasis [10], we propose that its ability to modulate ribosomal gene expression may represent an additional mechanism contributing to its therapeutic effects.

The present in vivo study indicates that SPD administration attenuates the progressive decline in muscle strength observed in ALS mice, suggesting a protective effect on motor performance and functional capacity. These findings are consistent with previous reports showing that exogenous SPD can also extend the survival in the SOD1-G93A mouse model of ALS [17]. Importantly, to favor potential translational relevance, SPD was administered as a dietary supplement through drinking water, a simple and non-invasive strategy that could be realistically applied in clinical settings. Increasing evidence supports the beneficial biological effects of spermidine across different experimental systems. In several model organisms, including Drosophila and mice, SPD supplementation has been associated with improved physiological performance, enhanced cognitive function, and increased lifespan [82,83,84]. Moreover, several clinical trials with humans have demonstrated that SPD is safe, well tolerated and effective in mitigating cognitive decline [11,84,85,86]. It is important to acknowledge that the SOD1-G93A model represents a specific ALS subtype characterized by pronounced oxidative stress and mitochondrial dysfunction. While SOD1 mutation accounts for a small percentage of familial cases, recent transcriptomic profiling of postmortem cortex samples from sporadic ALS patients has revealed that the largest molecular subgroup, “ALS-Ox” (61%), exhibits a pronounced signature of oxidative and proteotoxic stress, alongside SOD1 expression [87]. While our in vivo results are limited to this model, additional experiments on NSC34 cells expressing the TDP-43 Q331K mutation showed that spermidine improves mitochondrial function, restoring key bioenergetic parameters. These findings suggest that the beneficial effects of spermidine may extend beyond a single genetic form and could be relevant to a broader ALS population characterized by oxidative stress.

## 5. Conclusions

In conclusion, while further studies are needed to clarify the molecular mechanisms of SPD and its clinical relevance in neurodegenerative diseases, our findings support its beneficial effects in the SOD1-G93A model. Given the heterogeneity of ALS and the limited contribution of SOD1 mutations, caution is required in generalizing these results. However, complementary data in NSC34 cells expressing the TDP-43 Q331K mutation show that SPD improves mitochondrial function, suggesting that its effects may extend beyond a single genetic context. Further investigations across diverse ALS models, including sporadic forms, will be critical to establishing whether the beneficial effects of spermidine are reproducible across distinct pathological backgrounds, thereby strengthening its potential as a broadly applicable therapeutic strategy.

## Figures and Tables

**Table 1:** Deregulated mt-DNA encoded genes in gastrocnemius of SOD1-G93A mice. List of mtDNA genes down-regulated in GNM of SOD1-G93A mice, whose expression is mitigated by SPD supplementation. Values are expressed as Log2(FoldChange).

Gene Symbol	ALS vs. CTR Log2(FC)	ALS_SPD vs. ALS Log2(FC)
mt-Nd1	−1.66	1.48
mt-Nd2	−2.19	1.88
mt-Cox I	−1.16	1.16
mt-Cox II	−1.62	1.41
mt-Cox III	−1.11	0.83
mt-Atp8	−1.5	1.32
mt-Atp6	−1.62	1.36
mt-Nd3	−2.44	1.77
mt-Nd4l	−2.03	1.78
mt-Nd4	−1.53	1.54
mt-Nd5	−1.72	1.89
mt-Nd6	−2.09	2.27
mt-Cytb	−1.23	1.56

## Data Availability

FASTQ files can be retrieved from the SRA database at BioProject with accession code PRJNA1223828.

## Supplementary Materials

The following supporting information can be downloaded at https://www.mdpi.com/article/10.3390/biom16040566/s1; Supplementary Data S1–S6.xlsx. Supplementary Data S1–S6: RNAseq, ALSoD gene comparison and DEGs between conditions. Supplementary Tables and Figures.pdf. Supplementary Tables S1–S4: Number of Differentially Expressed Genes (DEGs) found in RNA-seq from both tissues; primer lists. Supplementary Figures S1–S3: MDS analysis; Mitochondrial-DNA copy number analysis; GSEA plots.

## Abbreviations

The following abbreviations are used in this manuscript:

ALS	Amyotrophic Lateral Sclerosis
ATP	Adenosine Triphosphate
AMPK	AMP-Activated Protein Kinase
CNS	Central Nervous System
DEG	Differentially Expressed Gene
DNA	Deoxyribonucleic Acid
ECM	Extracellular Matrix
EDTA	Ethylenediaminetetraacetic acid
ER	Endoplasmic Reticulum
FC	Fold Change
FCCP	Carbonyl Cyanide-p-Trifluoromethoxyphenylhydrazone
FDR	False Discovery Rate
FUS	Fused in Sarcoma
GNM	Gastrocnemius
GSEA	Gene Set Enrichment Analysis
HPLC	High-Throughput Liquid Chromatography
NES	Normalized Enrichment Score
OCR	Oxygen Consumption Rate
ODC	Ornithine Decarboxylase
ODC1	Ornithine Decarboxylase 1
ORA	Over-Representation Analysis
PA	Polyamine
PAOX	Polyamine Oxidase
PCR	Polymerase Chain Reaction
PGC	Peroxisome Proliferator-Activated Receptor Gamma Coactivator 1-Alpha
PUT	Putrescine
RNA	Ribonucleic Acid
ROS	Reactive Oxygen Species
SAT1	Spermidine/Spermine N1-Acetyltransferase
SC	Spinal Cord
SDS	Sodium Dodecyl Sulfate
SEM	Standard Error of the Mean
SIRT1	Sirtuin 1
SMOX	Spermine Oxidase
SMS	Spermine Synthase
SOD1	Superoxide Dismutase 1
SPD	Spermidine
SPM	Spermine
SPP1	Secreted Phosphoprotein 1
SRM	Spermidine Synthase
TDP43	TAR DNA-Binding Protein 43
TGFb	Transforming Growth Factor Beta
TORC1	Target of Rapamycin Complex 1
TYROBP	TYRO Protein Tyrosine Kinase Binding Protein
WT	Wild Type
