# Supplementary material for: Transcriptomic Analysis Reveals the Beneficial Effects of Spermidine in an ALS Mouse Model
Source: Biomolecules. 2026 Apr 10;16(4):566. doi: 10.3390/biom16040566 (PMC13113488; doi:10.3390/biom16040566)
Supplement: Supplementary file 1 [file biomolecules-16-00566-s001.zip › Supplementary Tables and Figures.pdf]

**Supplementary Table S1 – Number of Differentially Expressed Genes (DEGs) found in RNA-seq from both tissues**

| Tissue | Comparison | DEGs | lncRNA | | Protein-coding | |
|---|---|---|---|---|---|---|
| **SC** | **ALS vs. CTR** | 1215 | ↑14 | ↓7 | ↑1014 | ↓180 |
| | **ALS_SPD vs. ALS** | 14 | ↑1 | ↓0 | ↑1 | ↓13 |
| **GNM** | **ALS vs. CTR** | 7929 | ↑137 | ↓278 | ↑3769 | ↓3745 |
| | **ALS_SPD vs. ALS** | 4028 | ↑161 | ↓14 | ↑2090 | ↓1763 |

**Supplementary Table S2 – List of primer used for genotyping**

| **Target** | **Sequence** |
|---|---|
| Sod1 | Fwd: 5’- CATCAGCCCTAATCCATCTGA -3’<br>Rev: 5’- CGCGACTAACAATCAAAGTGA -3’ |
| Il-2 | Fwd: 5’- TAGGCCACAGAATTGAAAAGATCT -3’<br>Rev: 5’- GTAGGTGGAAATTCTAGCATCATCC -3’ |

**Supplementary Table S3 – List of primer used for qPCR analysis**

| Target | Sequence |
|---|---|
| Tgfb1 | Fwd 5'- CCCGAAGCGGACTACTATG -3'<br>Rev 5'- CACGGGACAGCAATGGGG -3' |
| Tgfbr2 | Fwd 5'- GTACTCTGGGAAATGACGTCCC -3'<br>Rev 5'- GTGGTTGAGCCAGAAGCTGG -3' |
| Sln | Fwd 5'- TGAGGTCCTTGGTAGCCTGA -3'<br>Rev 5'- CACACCAAGGCTTGTCTTCA -3' |
| Odc1 | Fwd 5'- CAGGATTTGACTGTG -3'<br>Rev 5' - TAACCCTCTCTGCAG -3' |
| Sms | Fwd 5'- ACAAGAATGGCAGCTTTGCC -3'<br>Rev 5' – GAACTATGGGTGGTAATCGC -3' |
| Srm | Fwd 5'- CGGAAGGTGCTGATCATCG -3'<br>Rev 5'- TCGCCCACGTGGAGAGT -3' |
| Sat1 | Fwd 5'- CACTGGACCCCTGAAGGTTA -3'<br>Rev 5'- CAGCAACTTGCCAATCCATG -3' |
| Smox | Fwd 5'- ACTCCAAGAATGGCGTGGC -3'<br>Rev 5'- CGACGCTGTTCTGACTCTC -3' |
| Paox | Fwd 5'- GGGAAGATACATCGCCCTTA -3'<br>Rev 5'- GGACCAAAAATCCAATGAGC -3' |
| B2m | Fwd 5'- ACAGTTCCACCCGCCTCACATT -3'<br>Rev 5'- TAGAAAGACCAGTCCTTGCTGAAG -3' |
| mt-Nd1 | Fwd 5'- ATGGCCATAGCCTTCCTAAC -3'<br>Rev 5'- GTTGTTAAAGGGCGTATTGGT -3' |
| mt-Nd2 | Fwd 5'- TACCCGCTACTCAACTCTAC -3'<br>Rev 5'- CATCCTATGTGGGCAATTGATG -3' |
| mt-Nd6 | Fwd 5'- TGTTAGTGGGTTTGTTGGTTG -3'<br>Rev 5'- CCCAAGTCTCTGGATATTCC -3' |
| Ppargc1a | Fwd 5'- AGAGTGTGCTGCTCTGGTTG -3'<br>Rev 5'- TTCCGATTGGTCGCTACACC -3' |
| Rpl3 | Fwd 5'- GGTGTTTGGGCAGGATGAGA -3'<br>Rev 5'- CCAGGCTCCAATACAGGCAA -3' |
| Rps14 | Fwd 5'- AGGAAACCATCTGCCGAGTG -3'<br>Rev 5'- GGTCCTGTTTCCTCCTGTGG -3' |
| Fabp7 | Fwd 5'- CAGTCAGGAAGGTGGCAAAGTG -3'<br>Rev 5'- GCTTGTCTCCATCCAACCGAAC -3' |
| S100a1 | Fwd 5'- AGGAAACCATCTGCCGAGTG -3'<br>Rev 5'- GTGAGATTCGTCAGATTCATCCG -3' |
| Spp1 | Fwd 5'- CTGGAGAGTGCCATGGAGAC -3'<br>Rev 5'- GTCCACAGCATCTGCATCCT -3' |
| Ly9 | Fwd 5'- CCACCAGGTACAGACAAGC -3'<br>Rev 5'- CGCTGCTCTCAGAGCTGG -3' |
| Gapdh | Fwd 5'- GGTTGTCTCCTGCGACTTC -3'<br>Rev 5'- GGTGGTCCAGGGTTTCTTAC -3' |

**Supplementary Table S4. List of primers used for mtDNA/nDNA ratio quantification**

| Target | Sequence |
|---|---|
| mt-Co1 | Fwd: 5'- TGCTAGCCGCAGGCATTAC -3'<br>Rev: 5'- GGTGCCCAAAGAATCAGAAC -3' |
| mt-Rnr2 | Fwd: 5'- CCGCAAGGGAAAGATGAAAG -3'<br>Rev: 5'- CGTTTGGTTTCGGGGTTTC -3' |
| B2m | Fwd: 5'- ATGGGAAGCCGAACATACTG -3'<br>Rev: 5'- CAGTCTCAGTGGGGGTGAAT -3' |
| Ndufv1 | Fwd: 5'- CTTCCCCACTGGCCTCAAG –'3<br>Rev: 5'- CCAAAACCCAGTGATCCAGC –'3 |

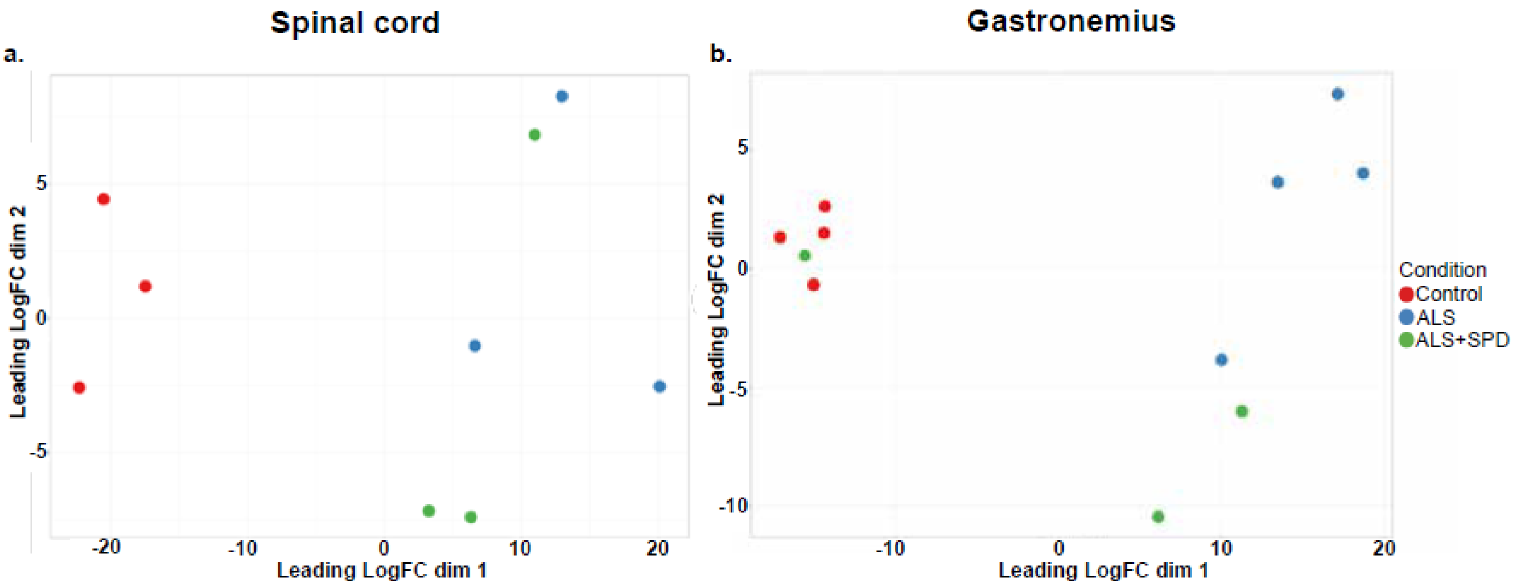


**Supplementary Figure S1**. MDS analysis for a) Spinal cord and b) Gastrocnemius.

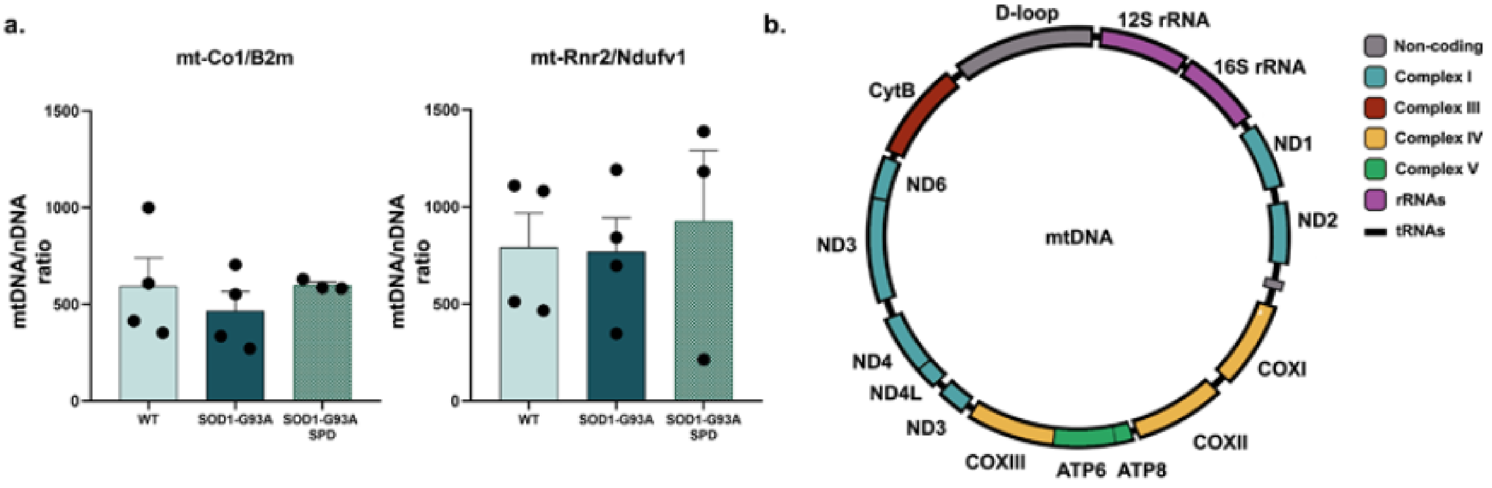


**Supplementary Figure S2.** Mitochondrial-DNA copy number analysis in GNM. a-b) qPCR on mt-DNA in GNM. Data are represented as mean ± SEM using expression levels with 2-ΔCt method for n=3 mice per experimental group. One-way ANOVA followed by Tukey post-hoc test were used.

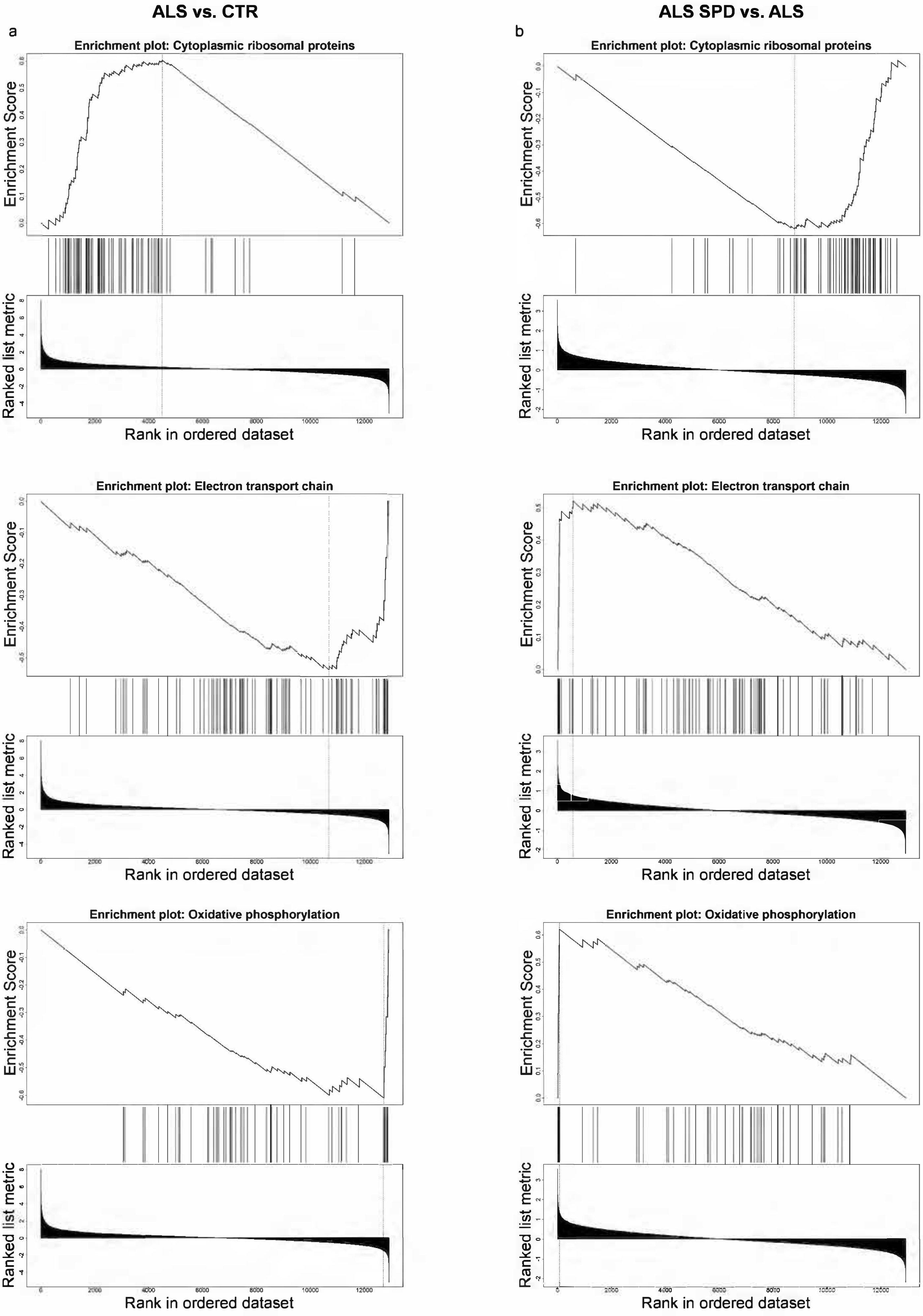


**Supplementary Figure S3**. GSEA plot for enriched pathways validated for Gastrocnemius. a) GSEA plot for ALS vs. CTR comparison and b) GSEA plot for ALS_SPD vs. ALS comparison
